# Analysis of natural female post-mating responses of *Anopheles gambiae* and *Anopheles coluzzii* unravels similarities and differences in their reproductive ecology

**DOI:** 10.1038/s41598-018-24923-w

**Published:** 2018-04-26

**Authors:** Janis Thailayil, Paolo Gabrieli, Beniamino Caputo, Priscila Bascuñán, Adam South, Abdoulaye Diabate, Roch Dabire, Alessandra della Torre, Flaminia Catteruccia

**Affiliations:** 1 Division of Cell and Molecular Biology, Imperial College London, Imperial College Road, London, SW7 2AZ United Kingdom; 2 Department of Immunology and Infectious Diseases, Harvard T. H. Chan School of Public Health, Boston, Massachusetts USA; 3 Dipartimento di Medicina Sperimentale, Università di Perugia, Perugia, Italy; 4 Dipartimento di Sanità Pubblica e Malattie Infettive, Laboratory affiliated to Istituto Pasteur Italia - Fondazione Cenci Bolognetti, Sapienza University of Rome, Rome, Italy; 5 Institut de Recherche en Sciences de la Santé (IRSS)/Centre Muraz, Bobo-Dioulasso, 01 BP 545 Burkina Faso; 6 Present Address: Dipartimento di Biologia e Biotecnologie, Università degli studi di Pavia, Pavia, Italy; 7 Present Address: Escuela de Microbiología, Universidad de Antioquia (UdeA), Medellìn, Colombia

## Abstract

*Anopheles gambiae* and *An. coluzzii*, the two most important malaria vectors in sub-Saharan Africa, are recently radiated sibling species that are reproductively isolated even in areas of sympatry. In females from these species, sexual transfer of male accessory gland products, including the steroid hormone 20-hydroxyecdysone (20E), induces vast behavioral, physiological, and transcriptional changes that profoundly shape their post-mating ecology, and that may have contributed to the insurgence of post-mating, prezygotic reproductive barriers. As these barriers can be detected by studying transcriptional changes induced by mating, we set out to analyze the post-mating response of *An. gambiae* and *An. coluzzii* females captured in natural mating swarms in Burkina Faso. While the molecular pathways shaping short- and long-term mating-induced changes are largely conserved in females from the two species, we unravel significant inter-specific differences that suggest divergent regulation of key reproductive processes such as egg development, processing of seminal secretion, and mating behavior, that may have played a role in reproductive isolation. Interestingly, a number of these changes occur in genes previously shown to be regulated by the sexual transfer of 20E and may be due to divergent utilization of this steroid hormone in the two species.

## Introduction

Although overall malaria mortality rates have significantly declined since 2010 due to increased prevention and control measures, Sub-Saharan Africa continues to carry a disproportionately high share of the global malaria burden, bearing more than 90% of the 212 million cases and of the estimated 429,000 deaths caused by *Plasmodium* parasites^1^. One of the main reasons for this higher burden in the African continent is the presence of a very efficient mosquito vectorial system, principally represented by the two most recently radiated species of the *Anopheles gambiae* complex, i.e. *Anopheles gambiae* and *An. coluzzii*^2,3^. These species are sympatric in West and Central Africa^4,5^, but differ in their larval ecology, with *An. gambiae* being more adapted to temporary rain-dependent and *An. coluzzii* to permanent anthropogenic breeding sites^6–9^. Due to their major role as malaria vectors, the two species are the target of several studies aimed at developing novel approaches for the control of disease transmission in sub-Saharan Africa, with the view to complement or strengthen current insecticide-based control methods^10–12^.

One of these novel approaches consists in manipulating the mosquito reproductive success. A recent study showed that application of non-steroidal agonists of the steroid hormone 20-hydroxyecdysone (20E) on *An. gambiae* virgin females virtually sterilizes them by preventing their insemination and reducing egg development^10^. This hormone is a potent regulator of gene transcription during both juvenile development and oogenesis in adults^13,14^, and in some anopheline species is synthetized in the male accessory glands (MAGs) and transferred during mating to the female atrium together with other seminal secretions embedded in a gelatinous structure named the mating plug^15–20^. Several studies have shown that in species of the Afrotropical *An. gambiae* complex, sexual transfer of 20E is essential for proper induction of female post-mating behaviors, such as refractoriness to further mating, enhanced egg production, triggered egg laying and increased fertility^17–19,21^. In addition, 20E injections in the G3 strain, which is a mixture of *An. gambiae* and *An. coluzzii*, induce a broad transcriptional response in the female reproductive tract, closely overlapping with the vast response induced by mating in the same strain, where hundreds of genes are up-and down-regulated at different time points after copulation^18,22^. However, the extent to which the response to mating is conserved between *An. gambiae* and *An*. *coluzzii* females is currently unknown.

In *Drosophila*, multiple lines of evidence point to a role of female post-mating biology in the insurgence of post-mating, prezygotic reproductive barriers. For instance, in crosses between recently diverged species, failure in sperm transfer and/or storage in hetero-specific crosses was attributed to mating-induced changes^23,24^. Additionally, processing of the insemination “plug” that forms in *Drosophila* females immediately after mating takes longer in hetero- than in homo-specific crosses^25,26^. It has also been postulated that fast evolving male-female molecular interactions or post-mating changes in transcript abundance may represent signatures of natural selection shaping the evolutionary arms race between the sexes^27–30^.

Post-mating events may have also played a role in the recent divergence between *An. gambiae* and *An. coluzzii*. While hybrid males from most crosses between species of the *An. gambiae* complex are sterile, males from crosses between *An. gambiae* and *An. coluzzii* do not show signatures of genetic incompatibilities and are fully fertile, with no obvious loss in fitness under laboratory conditions^31^. Nevertheless, hybrids between these two species are rarely observed in most areas of sympatry^5,32^. Where the two species are sympatric, e.g. in Burkina Faso, spatial and temporal segregation of the swarms is significantly contributing to assortative mating^33,34^, while close-range mate recognition cues, such as species-specific flight tones and/or contact cuticular pheromones, are believed to reinforce pre-mating isolation^35–41^. Inter-specific mating couples have, however, been repeatedly collected in the field^42,43^, suggesting the co-occurrence of intrinsic and/or extrinsic post-mating isolation mechanisms. While the latter have been shown to play a role^5^, intrinsic post-mating isolation mechanisms have never been investigated.

Here we report the first data on the transcriptional changes induced by mating in *An. gambiae* and *An. coluzzii* females captured in natural mating swarms from Burkina Faso. Our results corroborate previous data obtained under laboratory conditions^18,22^, allow the identification of factors potentially important for mating, fertility and reproductive success in each species, and provide novel insights on inter-specific differences that shape their reproductive ecology and may help unravel the mechanisms of their reproductive isolation.

## Results

### Collection of mating couples from natural swarms

**Figure 1:**
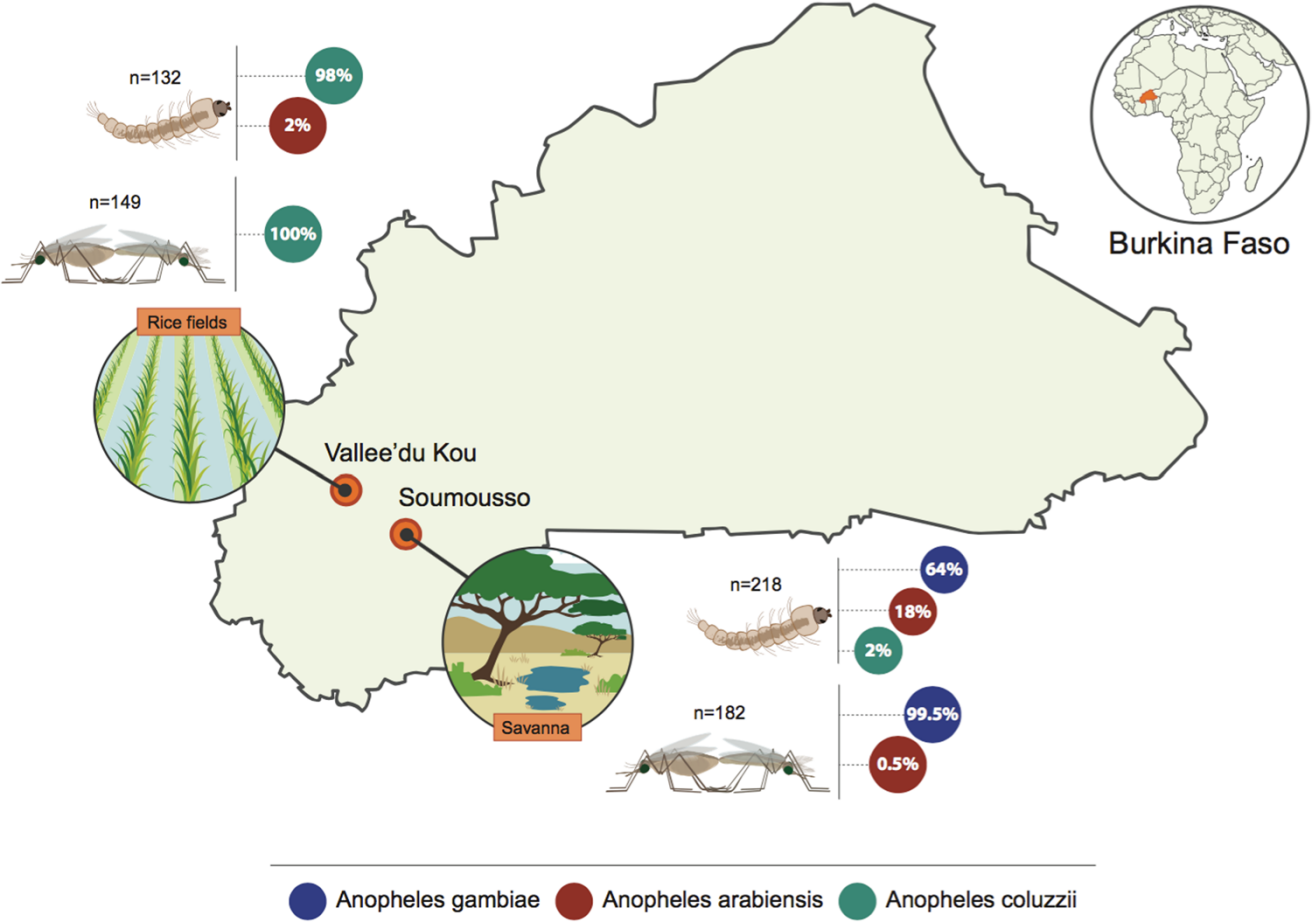
Schematic map of the collection sites in Burkina Faso. For each of the two sites (Vallèe du Kou and Soumousso), the total number of mosquitoes collected is indicated. The relative percentage of species is reported in the pie charts for both larval and adult samples, and species are color-coded as described in the figure. *Anopheles arabiensis* were not studied further. The map and the drawings have been generated using Illustrator CC 2017 (Adobe).

In order to analyze the natural post-mating response of females from the two anopheline sibling species, we collected 91 *An. gambiae* and 75 *An. coluzzii* mating couples from different swarms in the villages of Soumousso and Vallèe du Kou (Burkina Faso) (Fig. 1). Females of each couple were then dissected at either 1 day or 4 days post mating (PM), to capture the short-term as well as the lasting, long-term response to copulation. We dissected the lower reproductive tract (LRT) comprising atrium and spermatheca, and the rest of the body (carcass).

Virgin females were instead produced by collecting larvae from natural breeding sites, and LRTs and carcasses were dissected from resulting adult females at 2 and 5 days post emergence. Because the age of mated females could not be determined as they were caught in natural mating swarms, we chose these time points for tissue collection in virgins to approximately age-match these samples to the ones dissected from mated females, given that it is generally believed that females mate on the second night after emergence^44^.

### Post-mating transcriptional response in the lower reproductive tract (LRT) of field *An. gambiae* and *An. coluzzii* females

**Figure 2:**
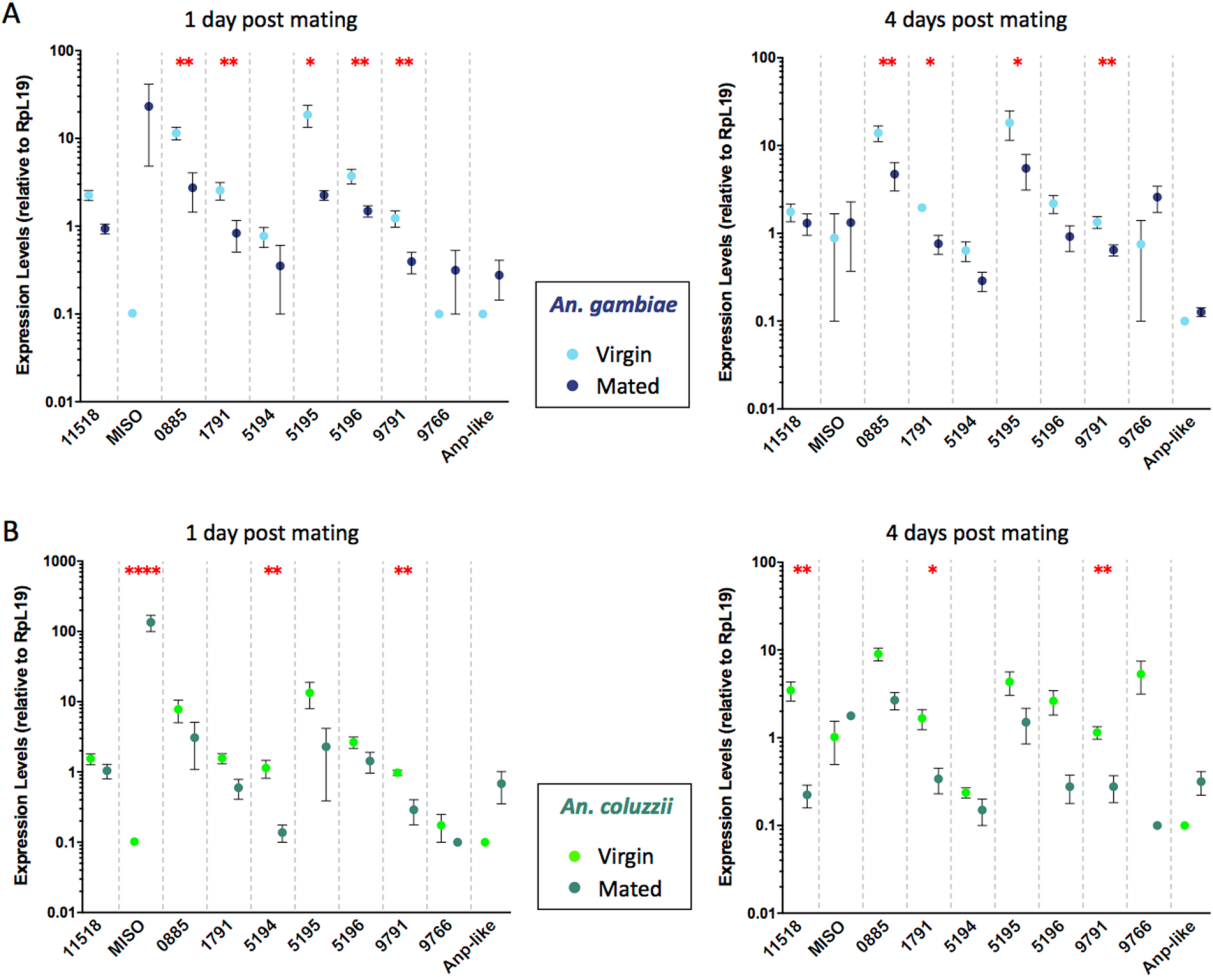
Gene expression levels after mating in the female lower reproductive tract (LRT). Gene expression levels are shown as Rpl19 normalized values (±Standard Error). (**A**) *An. gambiae* female LRT were tested as virgins (cyan dots) or at 1 day and 4 days post mating (blue dots). (**B**) *An. coluzzii* female LRT were tested as virgins (light green dots) or at 1 day and 4 days post mating (dark green dots). Red asterisks indicate a significant difference between mated and virgin females: *P < 0.05; **P < 0.01; ***P < 0.001; ****P < 0.001.

In our analysis of the post-mating response in the LRT, we focused on ten genes shown to be strongly up- or down-regulated after mating in laboratory experiments and thus likely to be involved in the reproductive processes triggered by copulation^18,22^ (Tables 1, 2; Fig. 2). These included 9 genes whose function in *An. gambiae* has not been determined yet – i.e. one ABC transporter (AGAP011518), three serine proteases (AGAP005194, AGAP005195, AGAP005196), one amino protease (AGAP000885), two metallopeptidases (AGAP001791 and AGAP009791), a protease inhibitor (AGAP009766), and a putative anti-microbial *Andropin-like* gene (AGAP009429)^45^. The last gene was the *mating induced stimulator of oogenesis* (*MISO*, AGAP002620), which is induced by the sexual transfer of the steroid hormone 20E and is implicated in the increase in egg development experienced by mated *An. gambiae* females after blood feeding^17^.

**Table 1:** *Anopheles gambiae* response to mating.

Tissue	Function	Gene	ANOVA P value	1 day post mating			4 days post mating		
				Virgin levels (mean ± SD)	Mated levels (mean ± SD)	Post-hoc Adj. P value	Virgin levels (mean ± SD)	Mated levels (mean ± SD)	Post-hoc Adj. P value
Lower Reproductive Tract	ABC transporter	*AGAP011518*	ns	2.256 ± 0.665	0.938 ± 0.245	ns	1.758 ± 0.809	1.307 ± 0.950	ns
	Oogenesis	*MISO*	ns	0.102 ± 0.005	23.18 ± 31.765	ns	0.885 ± 1.570	1.329 ± 2.534	ns
	Proteolysis	*AGAP000885*	**0.0038**	**11.508** ± **4.195**	**2.750** ± **2.61**	**0.0092**	**13.920** ± **5.626**	**4.726** ± **4.479**	**0.0085**
		*AGAP001791*	**0.0042**	**2.562** ± **1.298**	**0.835** ± **0.654**	**0.0096**	**1.960** ± **0.307**	**0.761** ± **0.494**	**0.0276**
		*AGAP005194*	ns	0.770 ± 0.396	0.353 ± 0.505	ns	0.6375 ± 0.320	0.289 ± 0.189	ns
		*AGAP005195*	**0.0179**	**18.590** ± **11.597**	**2.260** ± **0.504**	**0.0242**	**18.143** ± **13.357**	**5.507** ± **6.346**	**0.0368**
		*AGAP005196*	**0.0023**	**3.742** ± **1.583**	**1.493** ± **0.443**	**0.0056**	2.188 ± 1.004	0.919 ± 0.788	ns
		*AGAP009791*	**0.0030**	**1.232** ± **0.516**	**0.395** ± **0.216**	**0.0064**	**1.343** ± **0.418**	**0.646** ± **0.244**	**0.004**
	Protease inhibitor	*AGAP009766*	ns	0.100 ± 0.001	0.315 ± 0.430	ns	0.750 ± 1.300	1.307 ± 0.950	ns
	Other	*Andropin-like*	ns	0.100 ± 0.001	0.277 ± 0.229	ns	0.100 ± 0.001	0.127 ± 0.039	ns
Carcass	Behavior	*dsx*	**0.0408**	**0.031** ± **0.009**	**0.018** ± **0.009**	**0.014**	0.028 ± 0.005	0.0021 ± 0.006	ns
		*CPF3*	**0.0007**	**0.068** ± **0.053**	**0.009** ± **0.015**	**0.0013**	0.007 ± 0.006	0.001 ± 0.001	ns
		*AP-1*	**0.0040**	**0.014** ± **0.004**	**0.008** ± **0.002**	**0.0123**	**0.011** ± **0.005**	**0.007** ± **0.003**	**0.0342**
		*lingerer*	ns	0.030 ± 0.006	0.035 ± 0.010	ns	0.028 ± 0.006	0.025 ± 0.008	ns
		*GSTE2*	ns	0.006 ± 0.003	0.005 ± 0.006	ns	0.004 ± 0.001	0.006 ± 0.003	ns
		*OBP25*	**0.0037**	**0.007** ± **0.003**	**0.014** ± **0.009**	**0.0109**	0.004 ± 0.002	0.005 ± 0.003	ns
	Lipid Transport	*Vg*	**<0.0001**	**0.002** ± **0.001**	**0.867** ± **0.318**	**<0.0001**	0.001 ± 0.001	0.039 ± 0.038	ns
	Immunity	*TEP1*	**0.0297**	**0.074** ± **0.023**	**0.214** ± **0.071**	**0.0044**	0.145 ± 0.122	0.121 ± 0.041	ns
		*LRIM1*	ns	0.032 ± 0.010	0.131 ± 0.124	ns	0.087 ± 0.034	0.096 ± 0.075	ns
		*CEC1*	ns	0.258 ± 0.104	0.203 ± 0.033	ns	1.150 ± 1.090	0.331 ± 0.225	ns
		*CEC3*	ns	0.905 ± 0.561	2.189 ± 2.987	ns	1.225 ± 0.489	2.227 ± 1.182	ns
		*GAMB*	ns	0.091 ± 0.059	0.043 ± 0.013	ns	0.065 ± 0.021	0.090 ± 0.030	ns

Gene expression levels (normalized against Rpl19) in virgin and mated females (±Standard deviation) are indicated. Data show the results for both the 1 day and 4 days post mating response. One way ANOVA and pairwise post-hoc FDR - adjusted P values are also reported. In bold are genes showing significant post-mating regulation.

**Table 2:** *Anopheles coluzzii* response to mating.

Tissue	Function	Gene	ANOVA P value	1 day post mating			4 days post mating		
				Virgin levels (mean ± SD)	Mated levels (mean ± SD)	Post-hoc Adj. P value	Virgin levels (mean ± SD)	Mated levels (mean ± SD)	Post-hoc Adj. P value
Lower Reproductive Tract	ABC transporter	*AGAP011518*	**0.0069**	1.544 ± 0.619	1.042 ± 0.543	ns	**3.467** ± **2.075**	**0.223** ± **0.112**	**0.0022**
	Oogenesis	*MISO*	**<0.0001**	**0.102** ± **0.005**	**134.506** ± **78.151**	**<0.0001**	1.544 ± 0.619	1.042 ± 0.543	ns
	Proteolysis	*AGAP000885*	ns	7.792 ± 6.183	3.080 ± 4.471	ns	9.017 ± 3.991	2.677 ± 1.030	ns
		*AGAP001791*	**0.0443**	1.566 ± 0.582	0.596 ± 0.422	ns	**1.666** ± **1.131**	**0.340** ± **0.192**	**0.05**
		*AGAP005194*	**0.0042**	**1.138** ± **0.727**	**0.138** ± **0.075**	**0.0021**	0.237 ± 0.083	0.150 ± 0.071	ns
		*AGAP005195*	ns	13.390 ± 12.16	2.280 ± 4.231	ns	4.341 ± 3.403	1.505 ± 0.926	ns
		*AGAP005196*	ns	2.650 ± 1.105	1.430 ± 1.045	ns	2.630 ± 2.165	0.277 ± 0.170	ns
		*AGAP009791*	**0.0013**	**0.976** ± **0.192**	**0.290** ± **0.254**	**0.0069**	**1.150** ± **0.495**	**0.277** ± **0.163**	**0.0046**
	Protease inhibitor	*AGAP009766*	ns	0.174 ± 0.165	0.100 ± 0.001	ns	5.303 ± 5.689	0.100 ± 0.001	ns
	Other	*Andropin-like*	ns	0.100 ± 0.001	0.682 ± 0.739	ns	0.100 ± 0.001	0.317 ± 0.165	ns
Carcass	Behavior	*dsx*	ns	0.043 ± 0.023	0.049 ± 0.047	ns	0.025 ± 0.010	0.019 ± 0.005	ns
		*CPF3*	ns	0.039 ± 0.019	0.089 ± 0.169	ns	0.008 ± 0.007	0.011 ± 0.014	ns
		*AP-1*	ns	0.010 ± 0.012	0.017 ± 0.018	ns	0.008 ± 0.004	0.005 ± 0.002	ns
		*lingerer*	ns	0.025 ± 0.012	0.049 ± 0.073	ns	0.046 ± 0.014	0.058 ± 0.029	ns
		*GSTE2*	ns	0.032 ± 0.016	0.036 ± 0.027	ns	0.032 ± 0.013	0.032 ± 0.015	ns
		*OBP25*	**0.0128**	0.029 ± 0.021	0.043 ± 0.035	ns	0.008 ± 0.005	0.005 ± 0.002	ns
	Lipid Transport	*Vg*	ns	0.009 ± 0.003	0.152 ± 0.267	ns	0.004 ± 0.004	0.046 ± 0.078	ns
	Immunity	*TEP1*	ns	0.094 ± 0.033	0.125 ± 0.046	ns	0.133 ± 0.060	0.171 ± 0.054	ns
		*LRIM1*	ns	0.037 ± 0.016	0.038 ± 0.007	ns	0.048 ± 0.014	0.053 ± 0.017	ns
		*CEC1*	ns	0.124 ± 0.060	0.295 ± 0.245	ns	0.562 ± 0.670	0.487 ± 0.168	ns
		*CEC3*	**0.0237**	0.651 ± 0.491	0.575 ± 0.343	ns	1.260 ± 0.326	0.640 ± 0.205	ns
		*GAMB*	ns	0.056 ± 0.046	0.068 ± 0.056	ns	0.107 ± 0.053	0.059 ± 0.023	ns

Gene expression levels (normalized against Rpl19) in virgin and mated females (±Standard deviation) are indicated. Data show the results for both the 1 day and 4 days post mating response. One way ANOVA and pairwise post-hoc FDR - adjusted P values are also reported. In bold are genes showing significant post-mating regulation.

In *An. gambiae* females, changes in gene expression were detected in five proteases (ANOVA analysis; AGAP000885 *P* = 0.0038, AGAP001791 *P* = 0.0042; AGAP005195 *P* = 0.0179; AGAP005196 *P* = 0.0023; AGAP009791 *P* = 0.0030), all downregulated at 1 day PM (post-hoc analysis with FDR correction; AGAP000885 *P* = 0.0092, AGAP001791 *P* = 0.0096; AGAP005195 *P* = 0.0242; AGAP005196 *P* = 0.0056; AGAP009791 *P* = 0.0064). Moreover, four of these genes showed reduced expression levels also at 4 days PM (post-hoc analysis with FDR correction; AGAP000885 *P* = 0.0085, AGAP001791 *P* = 0.0276; AGAP005195 *P* = 0.0368; AGAP009791 *P* = 0.004) (Table 1, Fig. 2).

In *An. coluzzii* females, transcript levels were reduced for three proteases. AGAP001791 was downregulated at 4 days PM (ANOVA *P* = 0.04; post-hoc analysis with FDR correction *P* = 0.05), AGAP005194 at 1 day PM (ANOVA *P* = 0.0042; post-hoc analysis with FDR correction *P* = 0.0021), and AGAP009791 at both time points analyzed (ANOVA *P* = 0.0013; post-hoc analysis with FDR correction: 1 PM *P* = 0.0069; 4 days PM *P* = 0.0046). Furthermore, *MISO* was strongly up-regulated at 1 day PM (ANOVA *P* < 0.0001; post-hoc analysis with FDR correction *P* < 0.0001), while the ABC transporter AGAP011518 was downregulated at 4 days PM (ANOVA *P* = 0.0069; post-hoc analysis with FDR correction *P* = 0.0022) (Table 2, Fig. 2).

With the exception of AGAP009766 and *Andropin-like*, which were previously shown to be upregulated after mating, results were consistent with those obtained in the laboratory, showing that the transcriptional response to mating is mostly conserved after colonization^18,22^.

### Post-mating transcriptional response of genes related to reproduction, mating behavior and immunity in the carcass of field *An. gambiae* and *An. coluzzii* females

**Figure 3:**
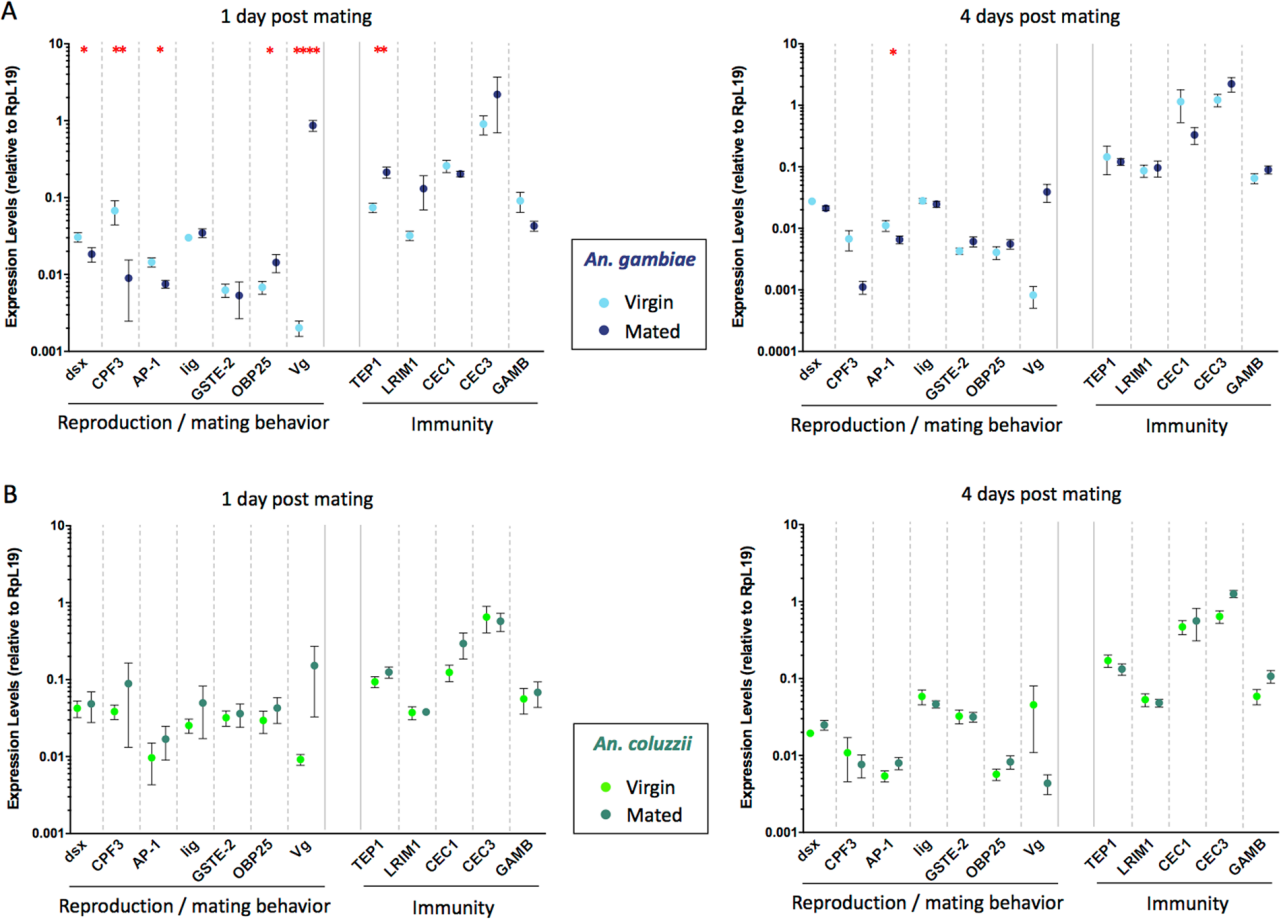
Gene expression levels after mating in the female carcass. Gene expression levels are shown as Rpl19 normalized values (±Standard Error). (**A**) *An. gambiae* female carcasses were tested as virgins (cyan dots) or at 1 day and 4 days post mating (blue dots). (**B**) *An. coluzzii* female carcasses were tested as virgins (light green dots) or at 1 day and 4 days post mating (dark green dots). Red asterisks indicate a significant difference between mated and virgin females: *P < 0.05; **P < 0.01; ***P < 0.001; ****P < 0.001.

We next analyzed the expression of genes in the female carcass, initially focusing on seven factors that may be related to reproductive success or mating behavior (Table 1 and Table 2; Fig. 3). Our analysis included *Vitellogenin* (*Vg*, AGAP004203), which encodes a yolk protein that is needed for egg development^46^ and which in *Aedes aegypti* mosquitoes is strongly upregulated by 20E synthetized after blood feeding^47^, and other six genes shown to be differentially expressed in *An. gambiae* and *An. coluzzii* virgin females^48^ that we reasoned might be associated with assortative mating behavior. These included: the sex determining gene *doublesex* (*dsx*, AGAP004050), the *antennal carrier protein AP-1*, (AGAP004799), the odorant binding protein 25 (*OBP25*, AGAP012320), the cuticular protein *CPF3* (AGAP004690), the *glutathione S transferases – epsilon class 2* (*GST-E2*, AGAP009194); and *lingerer* (AGAP004817). *dsx* regulates the terminal sexual differentiation in most insects^49^, and specifically it determines the differentiation of neurons that control male courtship behavior^50^ and female sexual receptivity^51–53^. In *Drosophila*, *lingerer* is involved in the control of male copulatory organs during courtship^54^. *CPF3* is a non-canonical cuticular protein with no chitin-binding capacity, which may be part of the epicuticle^55^ where it could bind to sex pheromones such as cuticular hydrocarbons (CHCs)^48^. Odorant binding proteins such as AP-1 and OBP25 transfer odorants to specific receptors^56^ and might play a role in female mate choice by helping the identification of co-specific males. GST-E2 might instead be involved in the metabolism of chemical stimuli from antennae and other sensory organs^57^, thus regulating the availability of stimulants such as CHCs.

While no changes were detected in *An. coluzzii* (Table 2, Fig. 3), in *An. gambiae* mean gene expression levels were different for five of the seven genes analyzed (*dsx* ANOVA *P* = 0.0408; *CPF3* ANOVA *P* = 0.0007; *AP-1* ANOVA *P* = 0.040; *OBP25* ANOVA *P* = 0.0037; *Vg* ANOVA *P* < 0.0001). *dsx*, *CPF3* and *AP-1* were downregulated at 1 day PM (post-hoc analysis with FDR correction *dsx P* = 0.014; *CPF3 P* = 0.0013; *AP-1 P* = 0.0123) and *AP-1* was down-regulated also at 4 days PM (*P* = 0.0342). *OBP25* and *Vg* were instead upregulated at 1 day PM (post-hoc analysis with FDR correction *OBP25 P* = 0.0109; *Vg P* < 0.0001) (Table 2, Fig. 3).

We finally tested whether mating induces a differential immune response in the two species, possibly driven by diverging sexually transmitted pathogens^58,59^. To this aim, we evaluated the expression levels in the female carcass of five immunity-related genes: the thioester containing protein 1 (*TEP1*), which is a complement-like factor, homologous to the human C3, that binds and mediates killing of pathogens including *Plasmodium* parasites^60^; the leucine-rich immune protein 1 (*LRIM1*), which circulates in the hemolymph as a disulphide-bounded complex with the leucine-rich protein APL1C and interacts with TEP1 controlling its activity^61,62^; and the antimicrobial peptides cecropin 1 (*CEC1*), *CEC3*, and gambicin (*GAMB*)^63,64^. Only *TEP1* showed to be upregulated in *An. gambiae* females at 1 day PM (ANOVA *P* = 0.0297; post-hoc test with FDR correction *P* = 0.0044).

## Discussion

Our results on the transcriptional response to mating in *An. gambiae* and *An. coluzzii* females collected from natural mating swarms largely corroborate previous data obtained under laboratory conditions^17,18,22^, demonstrating the opportunity of studying complex phenomena such as mating and post-mating behavior in laboratory colonies. This result is remarkable when considering that gene expression is age-dependent^65–67^ and that in our study it was not possible to precisely age-match mated females to virgin ones. For this reason - as well as for the limited number of samples we analyzed due to intrinsic difficulties in collecting couples from natural mating swarms - we observed some variability in our results that probably limited our power to detect subtler, age-dependent changes.

Despite these limitations, some interesting differences were detected in the post-mating responses of the two species samples. Although field *An. gambiae* and *An. coluzzii* males and females from the same geographic areas studied here share largely overlapping reproductive microbiomes^42^, we detected a mating-induced regulation of *TEP1*, a key immune gene, in the carcass of *An. gambiae* females. This species-specific upregulation may be due to sexual transfer of microorganisms populating the *An. gambiae* male reproductive tract, similarly to what observed in *D. melanogaster* where mating anticipates immune reactions to sexually transmitted pathogens possibly as a mechanism to enhance fecundity^68^.

Perhaps more interestingly, our data also highlight differential mating-induced changes in genes involved in oogenesis, which may reflect inter-specific differences in the physiological processes leading to egg development. First, we show that *MISO* – an atrial gene strongly induced by sexual transfer of 20E that regulates the number of eggs developed by females after mating and blood feeding^17,19^ – was significantly upregulated only in *An. coluzzii* at 1 day PM, although a trend towards an increase was also observed in *An. gambiae* at the same time point (Tables 1 and 2, Fig. 2). Given that MISO interacts in the atrium with 20E transferred during mating^17^, the differential transcriptional dynamics of this gene in the two species suggests that the timing of release of the steroid hormone from the mating plug may be regulated in a species-specific fashion. Second, we reveal that another 20E-induced gene important for oogenesis, *Vg*, is differentially regulated in the female carcass of the two anophelines. This yolk protein precursor, produced in the fat body and incorporated in the developing eggs via receptor-mediated endocytosis^69^, was strongly upregulated in *An. gambiae* at 1 day PM. This difference may reflect a reduced reliance of *An. coluzzii* females on mating for oogenesis, possibly due to an increased ability to store nutritional reserves during larval development^70^, and may provide some cues on why females of this species are competent to start egg development as virgins, while *An. gambiae* females generally need a mating-induced boost to promote the same process^44,70,71^. Even if the two species have a similar competence for *Plasmodium* transmission in the laboratory^72,73^, the fact that *An. gambiae* females often require multiple blood feedings to complete oogenesis^70^ may have important implication for malaria transmission in field settings, as it may increase its chances to become infected with *Plasmodium* parasites earlier in adult life and be associated with higher infection prevalence, as observed in some regions^74,75^.

We also detected differences in the regulation of the atrial proteolytic machinery which may be involved in the digestion of the mating plug and other seminal secretions. While the protease AGAP009791 was significantly repressed in both species at both time points analyzed, other proteases were downregulated in a time- and species-specific manner. Specifically, AGAP001791 was repressed at both time points in *An. gambiae* but only at 4 days PM in *An. coluzzii*; at 1 days PM AGAP000885, AGAP005195 and AGAP005196 were downregulated in *An. gambiae*, with AGAP000885 and AGAP005195 repressed also at 4 days PM in this species, while AGAP005194 levels were downregulated in *An. coluzzii* at 1 day PM only. As postulated for *MISO*, the differential expression of proteolytic enzymes is consistent with the occurrence of species-specific timing of digestion of seminal secretions, which is associated with fertility in *Drosophila* as well as *An. gambiae*^20,25,26^ and, when perturbed, can lead to speciation^23,24^. Intriguingly, several codons - including those close to the catalytic portion - of the genes encoding the atrial proteases AGAP005194, AGAP005195 and AGAP005196 are evolving under long-term and episodic positive selection in the *An. gambiae* complex^76^, supporting the hypothesis that timely and proper mating plug digestion might drive the emergence of post-mating pre-zygotic barriers in species of this complex. Similar to the activation of *MISO* and *Vg*, the post-mating downregulation of the proteolytic machinery appears to depend on the sexual transfer of 20E, as all six proteases analyzed here were repressed in the atrium of virgin laboratory females following 20E injection^18^.

Finally, the expression of four genes encoding for factors possibly involved in mating behavior (*dsx*, *CPF3*, *AP-1* and *OBP25*) was regulated by mating in *An. gambiae* females only. *dsx* is a key gene in the sexual differentiation cascade, and is produced as sex-specific isoforms^77,78^ that in *Drosophila* govern multiple aspects of reproductive biology, including the female receptivity to mating and the development and the activity of neural circuit that regulate sex-specific sexual behavior^51–53^. Furthermore, in the fruit fly *dsx* controls the expression of genes that synthetize female-specific long-chain cuticular hydrocarbons (CHC), notably the desaturase DESAT-F, that are potent pheromones for male courtship behavior^79^. It is therefore possible that *dsx* may affect the synthesis of CHC pheromones also in *An. gambiae* females, consistent with the observation that *An. gambiae* and *An. coluzzii* have, indeed, slightly different CHC profiles that are altered after mating^36,38^. Interestingly, *CPF3*, another gene related to CHC, is also downregulated in *An. gambiae* females. This cuticular protein is likely expressed in the epicuticle, where it is postulated to bind to cuticular pheromones such as CHCs^48^. Because post-mating changes in the CHCs profiles affect female attractiveness in many monandrous insect species^80,81^, the *An. gambiae*-specific downregulation of genes related to chemical contact cues might reflect the occurrence of different post-mating signals in the two species.

Interestingly, both cuticular proteins (CPs) and CHCs have been linked to 20E function, as this ecdysteroid reduces CP expression levels during development^82^ and is involved in CHC production in adult *Drosophila*^83^. Although a link between expression of the genes studied here and male-transferred 20E has yet to be confirmed in field setting, the different post-mating regulation of genes shown in laboratory conditions to be controlled by this steroid hormone^17,18,21^ supports the hypothesis of divergent male 20E effects in the two species, consistently with the finding of differential 20E levels in the MAGs of *An. coluzzii* and *An. gambiae* males in Burkina Faso^84^. It is intriguing to speculate that the transcriptional differences observed here could represent signatures of a divergent evolutionary arms race between the sexes, which in turn may have led to changes in key reproductive processes and possibly to the development of mechanisms of sexual isolation^85,86^.

## Materials and Methods

### Mosquito sample preparation

*Anopheles gambiae* and *An. coluzzii* were collected in September 2009 in the Western part of Burkina Faso, i.e. in the village of Soumousso (11°00′46′N, 4°02′45W) and in Vallèe du Kou (11°24′N, 4°24′W), located 55 km east and 30 km north-west of Bobo-Dioulasso, respectively. While in Soumousso larval breeding sites are mostly temporary, rain-dependent puddles more favorable to *An. gambiae*, the irrigation scheme in Vallèe du Kou largely favors *An. coluzzii*.

Virgin females were obtained from larvae collected in natural breeding sites (3 in Soumousso and 6 in Vallèe du Kou) and reared to the adult stage in natural climatic conditions and photoperiod using cages placed in the outdoor space available at the IRSS laboratory in Bobo-Dioulasso. In order to ensure females would not mate, pupae were individually transferred in single cups and their sex determined at emergence. Using this method, adult females and males were never in contact with each other.

Mating couples were collected from naturally occurring swarms as previously described^33,39,87^, allowed to complete copulation, transferred to single cups using mouth aspirators, and brought to the laboratory in sealed containers avoiding shifts in temperature and humidity.

Virgin and mated females were maintained in individual cups and DNA was extracted from single legs removed from live specimens for genotyping^88^ prior to dissections of reproductive organs. These were carried out under a dissecting stereo-microscope (5x magnification lens) at different time intervals, i.e. 2 and 5 days post emergence in the case of virgin females, and 24 hours and 4 days post-mating in the case of mated ones (to analyze both short-term and long-term response to mating). The time points for virgin females were selected to match as much as possible the age of mated females based on data showing that most females mate on the second night after emergence^44^. The lower reproductive tract (LRT, comprising atrium and spermathecae) and the rest of the body (carcass) of single females were stored separately in RNAlater solution (Ambion) and pools of five individual tissues/species/time interval were obtained for each time point (Table S1).

### RNA extraction and cDNA synthesis

For tissue-specific analysis, total RNA was extracted using TRI Reagent (Helena Biosciences). The amount of RNA for female carcasses was limited to 1 μg. All samples were treated with DNase I (Invitrogen), according to manufacturer’s guidelines. cDNAs were synthesized in 100 μl reactions using 1x First Strand buffer, 5 mM DDT, 0.5 mM dNTPs, 2.5 μM random hexamers, 40 units RNaseOut recombinant ribonuclease inhibitor, and 125 units of M-MLV reverse transcriptase (all reagents from Invitrogen).

### Quantitative Reverse Transcription PCR with SYBR green detection

Samples were run in 15 μl reaction volume using 1x Fast SYBR Green Master Mix (Applied Biosystems). Gene expression was quantified in duplicates on a StepOnePlus Real-Time thermocycler (Applied Biosystems) using the following program: 95 °C for 15 min, then 40 cycles (95 °C for 15 sec, 60 °C for 60 sec) followed by a dissociation curve analysis. Primers used for qRT-PCR are listed in Table S2. Three technical replicates were used for each biological replicate for each gene. A standard curve against serial dilutions of cDNA templates (mated and virgin) was used for each gene to determine the linear range of the assay.

### Statistical analysis

Gene expression levels were normalized using deltaCt method against the ribosomal gene *RpL*19 (AGAP004422), which is expressed at high levels and does not respond to mating^18,22^. To test for mating-induced changes in gene expression, the two species were studied separately. As we do not know if the primers anneal with the same efficiency or if the reference gene is expressed at the same levels in the two species, we did not perform cross-species comparisons. An ANOVA was first used to test whether each gene showed significant changes in the two time points analyzed, including in the analysis both mated and virgin samples. If the global F test gave positive results, pairwise *posthoc* contrast tests (Tukey-Kramer procedure) have been used to determine differences between mated and virgin females at each time point analyzed. To control for possible Type I error arising through use of multiple ANOVA tests, the *P* values were corrected by applying a False Discovery Rate procedure (FDR).
